# Natural ageing responses of duplex structured Mg-Li based alloys

**DOI:** 10.1038/srep40078

**Published:** 2017-01-05

**Authors:** C. Q. Li, D. K. Xu, B. J. Wang, L. Y. Sheng, Y. X. Qiao, E. H. Han

**Affiliations:** 1 CAS Key Laboratory of Nuclear Materials and Safety Assessment, Institute of Metal Research, Chinese Acadamy of Sciences, 62 Wencui Road, Shenyang 110016, China; 2 Key Laboratory for Anisotropy and Texture of Materials (Ministry of Education), Northeastern University, Shenyang 110819, China; 3 Peking University, Shenzhen Institute, Shenzhen Key Lab Human Tissue Regenerate & Repair, Shenzhen 518057, China; 4 Jiangsu University of Science & Technology, Zhenjiang 212003, China

## Abstract

Natural ageing responses of duplex structured Mg-6%Li and Mg-6%Li-6%Zn-1.2%Y alloys have been investigated. Microstructural analyses revealed that the precipitation and coarsening process of α-Mg particles could occur in β-Li phases of both two alloys during ageing process. Since a certain amount of Mg atoms in β-Li phases were consumed for the precipitation of abundant tiny MgLiZn particles, the size of α-Mg precipitates in Mg-6%Li-6%Zn-1.2%Y alloy was relatively smaller than that in Mg-6%Li alloy. Micro hardness measurements demonstrated that with the ageing time increasing, the α-Mg phases in Mg-6%Li alloy could have a constant hardness value of 41 HV, but the contained β-Li phases exhibited a slight age-softening response. Compared with the Mg-6%Li alloy, the age-softening response of β-Li phases in Mg-6%Li-6%Zn-1.2%Y alloy was much more profound. Meanwhile, a normal age-hardening response of α-Mg phases was maintained. Tensile results indicated that obvious ageing-softening phenomenon in terms of macro tensile strength occurred in both two alloys. Failure analysis demonstrated that for the Mg-6%Li alloy, cracks were preferentially initiated at α-Mg/β-Li interfaces. For the Mg-6%Li-6%Zn-1.2%Y alloy, cracks occurred at both α-Mg/β-Li interfaces and slip bands in α-Mg and β-Li phases.

Mg-Li alloys are the potential candidate lightweight structural materials in the fields of aerospace, electronics and biomedicine due to their low density, high specific strength, excellent formability and biodegradability^1^,^2^,^3^,^4^,^5^,^6^,^7^,^8^,^9^,^10^,^11^. Generally, Mg-Li alloys exhibit a typical duplex phase structure containing both α-Mg phase (Mg solid solution, HCP structure) and β-Li phase (Li solid solution, BCC structure) at room temperature when the addition of Li varies between 5.7 and 10.3 wt.%^12^,^13^. However, compared with the traditional Mg alloys, the mechanical strength of Mg-Li alloys is much lower even after severe plastic deformation (e.g. hot extrusion or equal channel angular extrusion)^1^,^2^,^14^,^15^,^16^. Moreover, their thermal stability is poor^17^,^18^,^19^,^20^, which is mainly ascribed to the early precipitation of phase particles during ageing process^17^,^18^,^19^,^20^,^21^,^22^,^23^. Wang *et al*. reported that the precipitation of α-Mg particles could easily occur in β-Li phases at room temperature and then cause the age-softening response of Mg-11%Li-1%Al-0.5%Zn alloy^17^. Moreover, Peng *et al*. found that the needle-shaped α-Mg particles could precipitate in β-Li phases of Mg-8%Li-3%Al-1%Y alloy during homogenization treatment at elevated temperature^18^. In addition, the metastable phase MgLi_2_X (X is Al or Zn) in the duplex Mg-Li based alloys could easily transform into soft equilibrium phase MgLiX during ageing process^14^,^19^,^20^,^21^,^22^,^23^. Qu *et al*. reported that the decrease in hardness for the LA96 alloy was mainly ascribed to the transformation from MgLi_2_Al phase into equilibrium phase AlLi at room temperature^19^. Similarly, for the Mg-Li-Zn system alloys, MgLi_2_Zn could also transform into MgLiZn and subsequently resulted in the age-softening responses at room temperature^22^.

As such, it is apparent that the poor thermal stability and notorious age-softening responses of Mg-Li alloys are evidently attributed to the α-Mg precipitates or phase transformation from MgLi_2_X to MgLiX. However, so far, rare systematic studies on the effects of α-Mg precipitates on the hardness and mechanical strength of duplex structured Mg-Li alloys have been reported. Recently, Xu *et al*. reported that I-phase (Mg_3_Zn_6_Y, icosahedral quasicrystal structure) could be *in-situ* formed through adding Zn and Y and controlling the Zn/Y atomic ratio of 6 in duplex structured Mg-Li alloys, which improved their mechanical strength to a great degree^2^,^14^,^15^,^16^. Additionally, I-phase exhibits many attractive properties such as high hardness, thermal stability, high corrosion resistance, low coefficient of friction and low interfacial energy, *etc*^24^,^25^,^26^,^27^,^28^,^29^. Therefore, the I-phase strengthened Mg-Li alloys could have excellent service properties and are the promising lightweight structural materials, but research work on their natural ageing behavior is limited. In this work, the target is to investigate and compare the microstructural evolution and natural ageing responses of Mg-6%Li and I-phase strengthened Mg-6%Li-6%Zn-1.2%Y alloys to answer the following three questions. (1) Whether the precipitation of α-Mg and/or equilibrium MgLiX precipitates could occur in β-Li phases of two alloys at room temperature or not? (2) If yes, which dominates the age-softening response? And (3) what is the mechanistic influence of precipitation on the mechanical strength of duplex Mg-Li alloys?

## Results and Discussion

### Microstructural characterization

**Figure 1:**
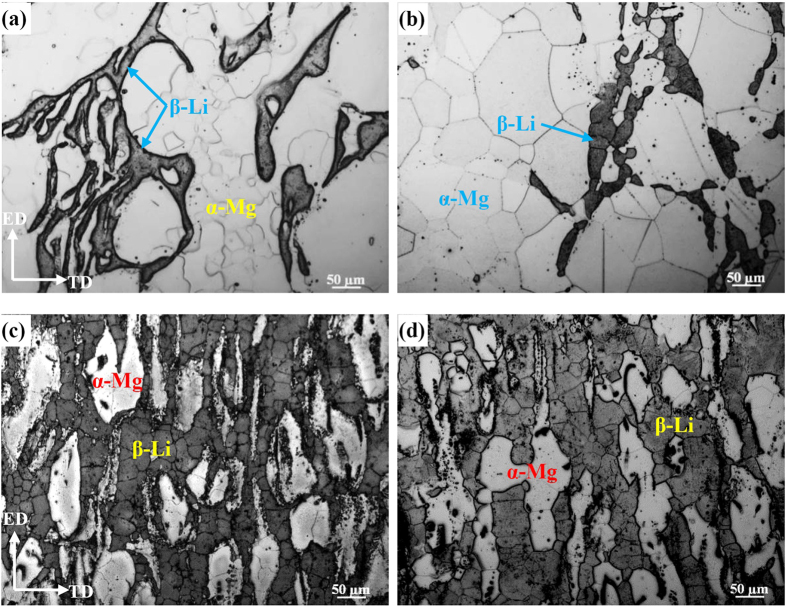
Optical observations to the etched surfaces of: (**a**) as-extruded and (**b**) solid solution treated samples of Mg-6%Li alloy, (**c**) as-extruded and (**d**) solid solution treated samples of Mg-6%Li-6%Zn-1.2%Y alloy.

Figure 1 shows the etched microstructure of two alloys before and after solid solution treatment. For the Mg-6%Li alloy, a typical duplex phase structure of (α-Mg + β-Li) can be observed (Fig. 1(a,b)). After solid solution treatment, the volume fraction of β-Li phases reduces and the average grain size of β-Li phases is 40 μm. Meanwhile, the initially inhomogeneous grains structure (with grain size ranging from 10 μm to 200 μm) of α-Mg phases becomes quite uniform with an average grain size of 120 μm. However, for the Mg-6%Li-6%Zn-1.2%Y alloy, an obviously higher volume fraction of β-Li phases was formed and preferentially distributed along the extrusion direction. After solid solution treatment, the grain structures of β-Li and α-Mg phases coarsened slightly (Fig. 1(c,d)). For the β-Li phases, the grain size increased from 26 μm to 35 μm. For the α-Mg phases, the grain size increased from 30 μm to 40 μm. Thus, it demonstrates that the addition of Zn and Y in Mg-6%Li-6%Zn-1.2%Y alloy can effectively impede the phase transformation from β-Li to α-Mg and retard the grain growth. Similar results have also been reported in previous work^14^,^16^.

**Figure 2:**
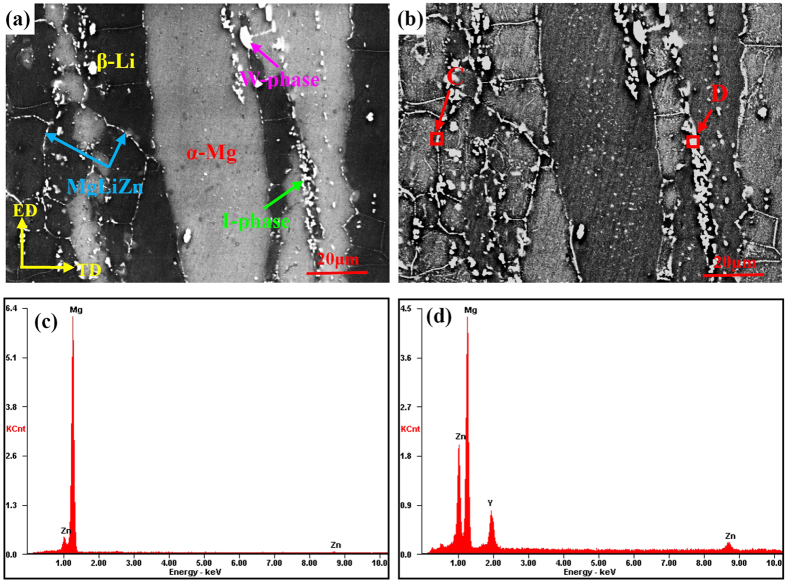
(**a**) Secondary electron and (**b**) backscattered electron images of the as-extruded Mg-6%Li-6%Zn-1.2%Y alloy; Images (**c**) and (**d**) are the SEM-EDS results of the marked areas of “C” and “D” in image (**b**).

Figure 2 shows the secondary electron (SE) and backscattered electron (BSE) images of the as-extruded Mg-6%Li-6%Zn-1.2%Y alloy. Moreover, the relevant EDS results to the selected phase particles are also provided. Due to the low atomic number, Li cannot be detected by EDS. Therefore, only elements of Mg and Zn were detected from the precipitates in β-Li phases (Fig. 2(c)). Meanwhile, the broken phases distributed along the extrusion direction are composed of Mg, Zn and Y (Fig. 2(d)). In the previous work, Xu *et al*. reported that the finely dispersed particles were MgLiZn particles and could quickly precipitate and coarsen in β-Li phases during artificial ageing at 200 °C^14^. Moreover, it demonstrated that the I-phase/α-Mg and W-phase/α-Mg eutectic pockets could be easily broken during extrusion process^2^,^16^. Additionally, the large bulk phases can be recognized as W-phase because they are hardly deformed during severe plastic deformation^16^,^30^. Then, the main phases existed in the as-extruded Mg-6%Li-6%Zn-1.2%Y alloy were labeled, as indicated in Fig. 2(a,b). Moreover, Zhang *et al*. reported that β-Li (BCC, Mg solute in Li solution) and α-Mg (HCP, Li solute in Mg solution) phases in duplex structured Mg-Li alloys exhibited black and gray in the secondary electron (SE) or backscatter electron (BSE) images, respectively^31^. However, the β-Li and α-Mg phases in the currently investigated Mg-6%Li-6%Zn-1.2%Y alloy were gray and black in BSE images, respectively. Generally, it is well known that the phases containing heavier elements exhibit brighter contrast in BSE images. In this work, most of element Y were consumed by the formation of I-phase and W-phase^31^,^32^,^33^,^34^, resulting in a very low solid solubility of Y in β-Li and α-Mg phases. However, Zhang *et al*. indicated that the solid solubility of Zn in β-Li phases was much higher than that in α-Mg phases at room temperature^31^. Actually, the presence of abundant MgLiZn precipitates in β-Li phases further confirms that the Zn solute in β-Li phases is much more than that in α-Mg phases. Therefore, it can be predicted that for the Mg-6%Li-6%Zn-1.2%Y alloy, the gray β-Li phases exhibited in the BSE image could be ascribed to their higher content of heavy element Zn.

### Microstructure evolution during ageing treatment

**Figure 3:**
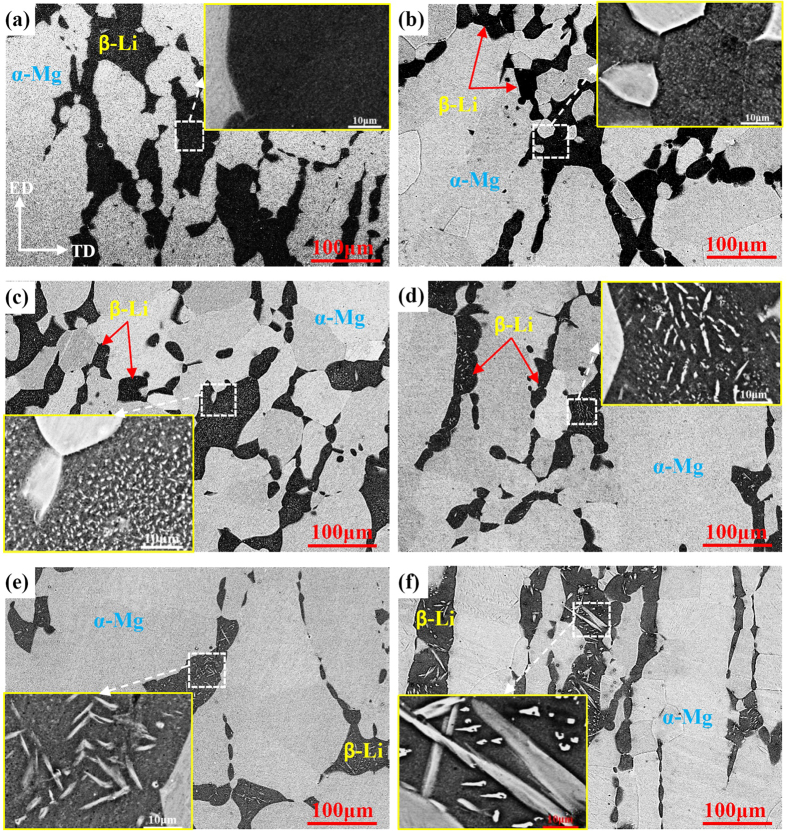
Microstructural evolution of the Mg-6%Li alloy subjected to natural ageing for (**a**) 0 days, (**b**) 7 days, (**c**) 21 days, (**d**) 35 days, (**e**) 42 days and (**f**) 180 days; High magnification observations to the squared area are inserted.

Figure 3 shows the microstructural evolution of Mg-6%Li alloy subjected to natural ageing treatment. It can be seen that the volume fraction and distribution of both α-Mg and β-Li phases have no obvious changes at a low magnification. Moreover, when the samples were observed immediately after solid solution treatment, no precipitation was found in β-Li phases even at high magnification, as shown in Fig. 3(a). However, with prolonging the ageing time, the precipitation and coarsening of phase particles could occur in β-Li phases. Therefore, it demonstrates that the β-Li phases are metastable. Since the duplex Mg-6%Li alloy only contains the elements of Mg and Li, the precipitates in β-Li phases are the α-Mg particles. Similarly, previous work also reported that needle-shaped α-Mg particles could precipitate in β-Li phases during ageing treatment^17^,^18^,^32^.

In general, a commonly accepted mechanism is that the precipitates nucleate at grain boundaries or vacancies, and grow up by the migration of reaction front, for which the driving force is the concentration difference of solute atoms between two sides of reaction front^35^. Additionally, the coarsening of precipitates could be induced by diffusional element transfer from small precipitates with high interfacial curvatures to large precipitates with low interfacial curvatures^36^. In this work, samples were quickly quenched into the room temperature water, which resulted in a high concentration of vacancies in the interior of matrix^37^. Then, these vacancies will play a key role in precipitation, especially at the early natural ageing stage. After natural ageing for 7 days, lots of tiny α-Mg particles were formed (Fig. 3(b)). When the ageing time increased to 21 days, obvious growth of the precipitated α-Mg particles occurred (Fig. 3(c)). Therefore, the dominant kinetics at the early natural ageing stage is the nucleation and growth of α-Mg precipitates. With the ageing time increasing, these tiny α-Mg precipitates will grow obviously and merge with each other (Fig. 3(d–f)). When the samples were naturally aged for 35 days, the size of some bigger precipitates was closed to 10 μm in length (Fig. 3(d)). When the samples were naturally aged for 42 days, all the α-Mg precipitates exhibited a uniform needle-shaped morphology with the size of about 10 μm in length (Fig. 3(e)). With the ageing time being extended to 180 days, some needle-shaped α-Mg precipitates became approximately lath-shaped morphology and their size reached 100 μm in length (Fig. 3(f)). Previous work reported that the growth and coarsening kinetics process of precipitates are mainly dependent on the diffusivity of alloying elements^36^,^38^. For the Mg-6%Li alloy, the loss of Li atoms in β-Li phases occurred easily because less volume fraction of β-Li phases was retained after solid solution treatment (Fig. 1(a,b)), which is consistent with the reported results^14^. Moreover, the solid solubility of Mg atoms in β-Li phases is higher at elevated temperature than that at room temperature. Due to the quick water quenching directly after solid solution treatment, the supersaturated Mg atoms in β-Li phases can be retained. Thus, the supersaturated β-Li phases became metastable at room temperature, resulting in the occurrence of nucleation, growth and coarsening of α-Mg precipitates in β-Li phases. Based on the above analysis, the kinetics of precipitated α-Mg particles should be determined by the diffusivity of Mg and Li atoms in β-Li phases.

**Figure 4:**
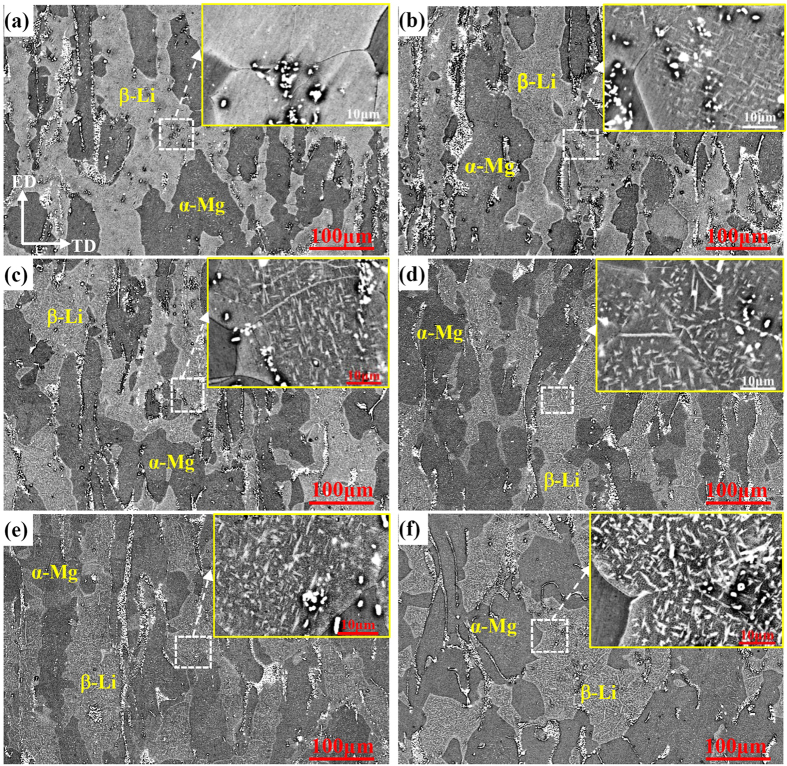
Microstructural evolution of the Mg-6%Li-6%Zn-1.2%Y alloy subjected to natural ageing for (**a**) 0 days, (**b**) 7 days, (**c**) 21 days, (**d**) 35 days, (**e**) 42 days and (**f**) 180 days; High magnification observations to the squared area are inserted.

**Figure 5:**
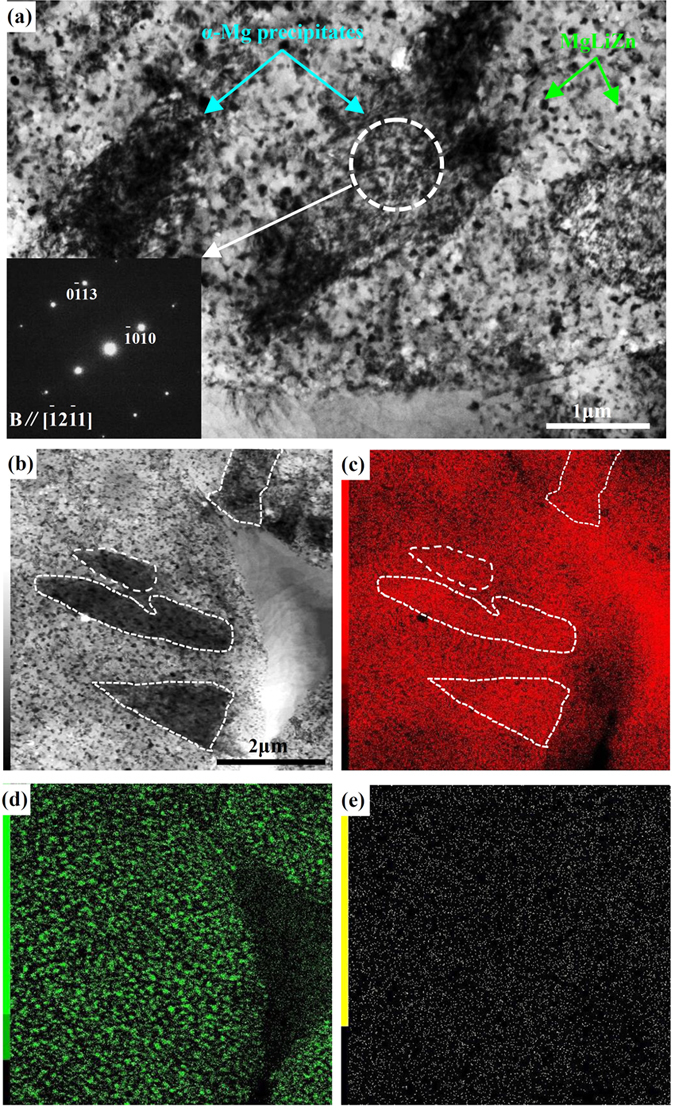
TEM morphologies and TEM-EDS elemental mappings of Mg-6%Li-6%Zn-1.2%Y alloy subjected to natural ageing for 7 days: (**a**) and (**b**) bright field images; The selected area diffraction patterns (SADPs) to the α-Mg precipitates is inserted in image (**a**); TEM-EDS elemental mappings of: (**c**) Mg, (**d**) Zn and (**e**) Y.

For comparison, ageing kinetics of precipitated particles in the I-phase reinforced Mg-6%Li-6%Zn-1.2%Y alloy was also observed, as shown in Fig. 4. Similar to the Mg-6%Li alloy, no precipitates in β-Li phases of Mg-6%Li-6%Zn-1.2%Y alloy can be observed immediately after solid solution treatment (Fig. 4(a)). After being naturally aged for up to 21 days, needle-shaped precipitates were formed in β-Li phases and their volume fraction is much more than that in Mg-6%Li alloy (Fig. 4(b)). However, with the ageing time increasing, the volume fraction of precipitates slightly increased and the coarsening of the needle-shaped precipitates was hardly observed. Even aged for 180 days, most precipitates still kept a relatively smaller size of less than 10 μm in length and 2 μm in width (Fig. 4(e)). To characterize the needle-shaped precipitates existed in β-Li phases of Mg-6%Li-6%Zn-1.2%Y alloy, TEM analysis and EDS elemental mapping were carried out for the sample being aged for 7 days, as shown in Fig. 5. It reveals that the diffraction patterns from the needle-shaped precipitates in β-Li phases could be indexed according to a hexagonal structure with lattice parameters of a = 0.3273 nm and c = 0.5221 nm, which is approximately consistent with lattice parameters of α-Mg phases in Mg-Li alloys^39^. Moreover, due to the low atomic number of Li, EDS mappings can only detect the existence of Mg, Zn and Y (Fig. 5(c–e)). It reveals that the needle-shaped precipitates were rich in Mg as indicated by the dotted lines (Fig. 5(c)). Therefore, it confirms that the needle-shaped particles were α-Mg precipitates. Meanwhile, lots of tiny particles containing a higher Zn concentration can also be observed in β-Li phases (Fig. 5(d)). Since the MgLiZn particles could rapidly precipitate and coarsen in β-Li phases^14^, these tiny particles should be MgLiZn precipitates. Moreover, the Zn-rich particles can easily precipitate in β-Li phases. Thus, it further clarifies the abnormal phenomenon that the β-Li and α-Mg phases in Mg-6%Li-6%Zn-1.2%Y alloy were gray and black in the BSE image (Fig. 2). Moreover, the different contrast between α-Mg phases and α-Mg precipitates should be ascribed to the contained MgLiZn particles in α-Mg precipitates (Fig. 5(a,b)). Additionally, it demonstrates that Y content in the matrix is very low (Fig. 5(e)). Thus, the effect of Y on the precipitation of needle-shaped α-Mg and/or MgLiZn could be neglected.

As for the ageing kinetics of precipitates in Mg-6%Li-6%Zn-1.2%Y alloy, the detailed discussion is as follows. Zhou *et al*. reported that the diffusivity of Zn is faster than that of Mg because the diffusion activation energy of transition metallic elements with *d* electrons is higher^38^. Therefore, the formation of MgLiZn precipitates should proceed quickly. Since the formed MgLiZn particles could act as nucleation sites, the nucleation kinetics of needle-shaped α-Mg precipitates in Mg-6%Li-6%Zn-1.2%Y alloy is faster than that in Mg-6%Li alloy at the initial ageing stage (Figs 3(b) and 4(b)). Meanwhile, it seems that the slower growth kinetics of α-Mg precipitates at the middle and late ageing stages is also related to the formed MgLiZn precipitates. On the one hand, due to the existed MgLiZn precipitates in β-Li phases, the barrier for the diffusion of Mg atoms in Mg-6%Li-6%Zn-1.2%Y alloy is much higher than that in Mg-6%Li alloy. On the other hand, previous work reported that the formation of I-phase can stimulate the formation of more β-Li phases in duplex Mg-Li based alloys^16^. After solid solution treatment, the volume fraction of β-Li phases in Mg-6%Li alloy decreased obviously, whereas the volume fraction of β-Li phases in Mg-6%Li-6%Zn-1.2%Y alloy was almost retained (Fig. 1). As such, it can be predicted that the Mg content in β-Li phases of Mg-6%Li-6%Zn-1.2%Y alloy should be higher than that of Mg-6%Li alloy. Since most Mg atoms in Mg-6%Li-6%Zn-1.2%Y alloy were consumed for the formation of abundant MgLiZn particles (Fig. 5), the decreased Mg content can hardly provide the driving force for the growth of α-Mg precipitates.

### Age-hardening behavior and tensile properties

**Figure 6:**
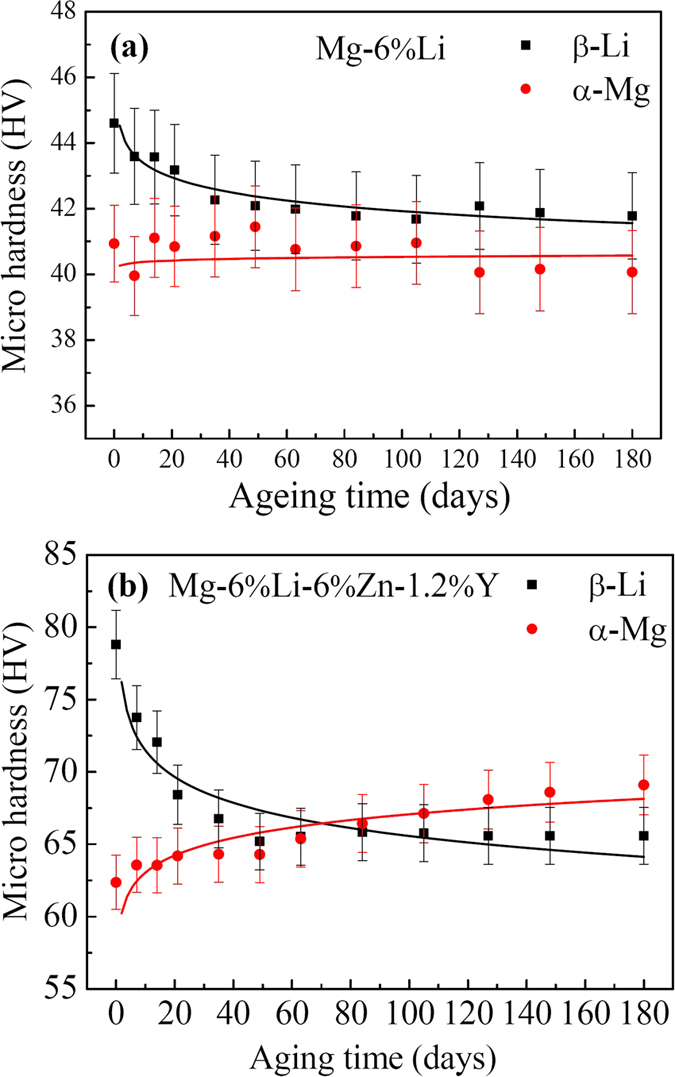
Micro hardness of β-Li and α-Mg phases versus natural ageing time curves for: (**a**) Mg-6%Li and (**b**) Mg-6%Li-6%Zn-1.2%Y alloys.

Figure 6 shows the micro Vickers hardness versus natural ageing time curves of β-Li and α-Mg phases in two alloys. It reveals that the hardness of β-Li phases in Mg-6%Li alloy exhibits an age-softening trend and reduces about 3 HV after natural ageing for 35 days (Fig. 6(a)). With further increasing the ageing time, the hardness of β-Li phases almost was stabilized at a value of 42 HV. However, the α-Mg phases maintain a constant hardness value of 41 HV. For the Mg-6%Li-6%Zn-1.2%Y alloy, the hardness value of β-Li phases decreases from 78 HV to 67 HV after natural ageing for up to 35 days (Fig. 6(b)). With further increasing the ageing time, the hardness of β-Li phases stabilizes at a value of 65 HV. Meanwhile, a normal age-hardening response of α-Mg phases is maintained and their hardness value can even be higher than that of β-Li phases when the ageing time exceeded 70 days. Based on the microstructural evolution (Fig. 3), the age-softening response of β-Li phases in Mg-6%Li alloy is ascribed to the formation and coarsening of α-Mg precipitates, which is consistent with the reported results^17^. However, the more hardness reduction of β-Li phases in Mg-6%Li-6%Zn-1.2%Y alloy is attributed to the formation of abundant MgLiZn particles and α-Mg precipitates (Figs 4 and 5).

In general, the change in the hardness values under different ageing conditions should be determined by the evolution of precipitated particles^37^,^40^. In the research of ageing behavior of Mg-Li-Zn alloy, Li *et al*. reported that the age-softening can easily occur even at the ageing temperature below 50 °C due to the phase transformation from MgLi_2_Zn to MgLiZn^22^. Moreover, Xu *et al*. reported that the precipitation and growth process of MgLiZn particles in β-Li phases can easily occur due to the high concentration of Li in β-Li phases and the fast mobility of Li atoms, leading to an obvious age-softening response of β-Li phases^14^. Therefore, the variation of hardness is due to the nucleation and growth of precipitates. In this work, MgLiZn and needle-shaped α-Mg precipitates can co-exist in Mg-6%Li-6%Zn-1.2%Y alloy (Figs 4 and 5). Previous work reported that the α-Mg precipitates could cause the ageing-softening response of Mg-Li alloys^14^,^17^. For the Mg-6%Li alloy, the precipitation of α-Mg can only induce a 3 HV hardness decrease of β-Li phases. Thus, the significant hardness decrease of β-Li phases in Mg-6%Li-6%Zn-1.2%Y alloy should be mainly ascribed to the formation of soft MgLiZn precipitates. Moreover, the α-Mg phases in Mg-6%Li-6%Zn-1.2%Y alloy present a normal age-hardening response with the ageing time increasing, which can be explained by the formation of metastable MgZn precipitates and/or GP zones during ageing treatment at the temperature below 100 °C^14^,^41^. Previous work demonstrated that the hardness of α-Mg and β-Li phases increases with the addition of alloying elements in duplex Mg-Li(-Zn/Al-Y) alloys and the hardness value of β-Li phases is higher than that of α-Mg phases^14^,^21^,^33^. Therefore, the higher hardness values of α-Mg and β-Li phases in Mg-6%Li-6%Zn-1.2%Y alloy should be ascribed to the addition of Zn and Y. Moreover, the formed phase particles (W-phase and I-phase) in the matrix can also contribute to the increase of hardness values.

**Figure 7:**
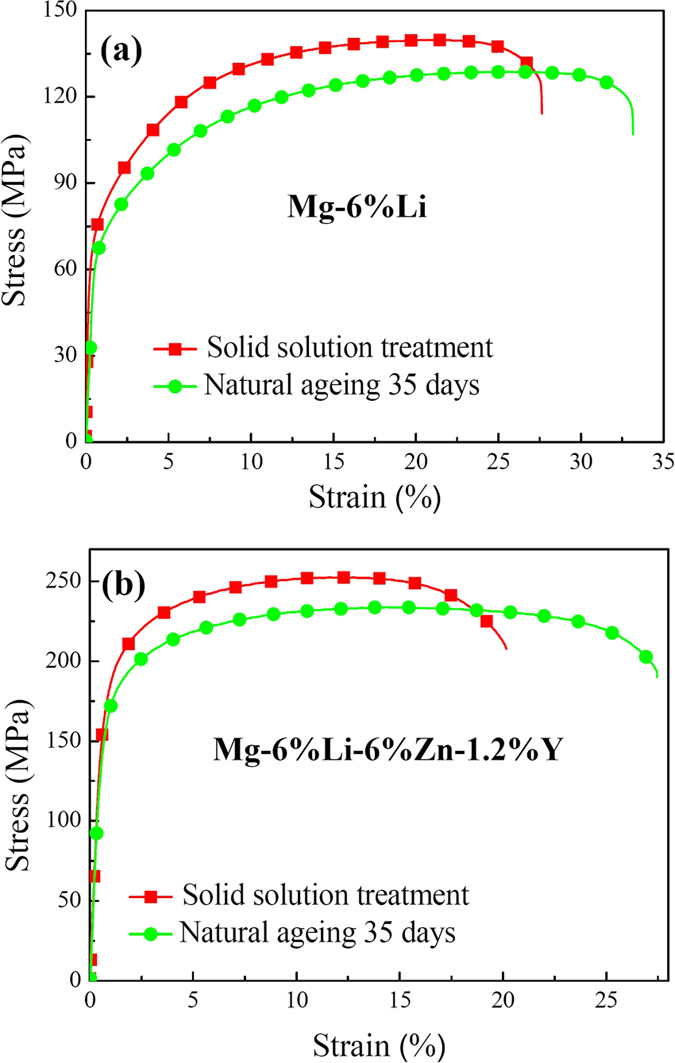
Tensile stress-strain curves of: (**a**) Mg-6%Li and (**b**) Mg-6%Li-6%Zn-1.2%Y alloys before and after natural ageing for 35 days.

It is commonly accepted that mechanical strength is positive proportional to the hardness value of the alloys^40^. Figure 7 shows the tensile stress-strain curves of two alloys before and after ageing treatment for 35 days. To describe and compare the measured data conveniently, the mechanical properties of 0.2% proof yield strength (YS), ultimate tensile strength (UTS) and elongation ratio to failure (EL) of two alloys are listed in Table 1. It can be seen that after solid solution treatment, the YS, UTS and EL values of the Mg-6%Li alloy are 80 MPa, 140 MPa and 27%, respectively. After ageing for 35 days, the YS and UTS values respectively reduce to 77 MPa and 133 MPa, but the EL value increases to 30%. For the Mg-6%Li-6%Zn-1.2%Y alloy immediately after solid solution treatment, the YS, UTS and EL values are 183 MPa, 252 MPa and 20%, respectively. After ageing for 35 days, the YS and UTS values of the Mg-6%Li-6%Zn-1.2%Y alloy respectively reduce to 171 MPa and 234 MPa, but the EL value increases to 27%. It can be seen that obvious age-softening responses can occur in both two alloys, which is consistent with the tendency of measured hardness values (Fig. 6). It has been reported that I-phase can have semi-coherent interfaces with the α-Mg matrix by introducing steps and ledges periodically along the interface, resulting in a strong atomic bonding of I-phase/α-Mg interfaces^42^. Previous work demonstrated that the I-phase/α-Mg interfaces can effectively retard the basal slip of dislocations^43^. Additionally, Xu *et al*. reported that I-phase is very effective for the strength improvement of Mg-Li alloys and effectively retard the transformation from β-Li to α-Mg in the duplex Mg-Li alloys at elevated temperature^14^,^44^. Thus, compared with Mg-6%Li alloy, the higher mechanical strength of Mg-6%Li-6%Zn-1.2%Y alloy can be ascribed to the formation of I-phase.

### Failure analysis

**Figure 8:**
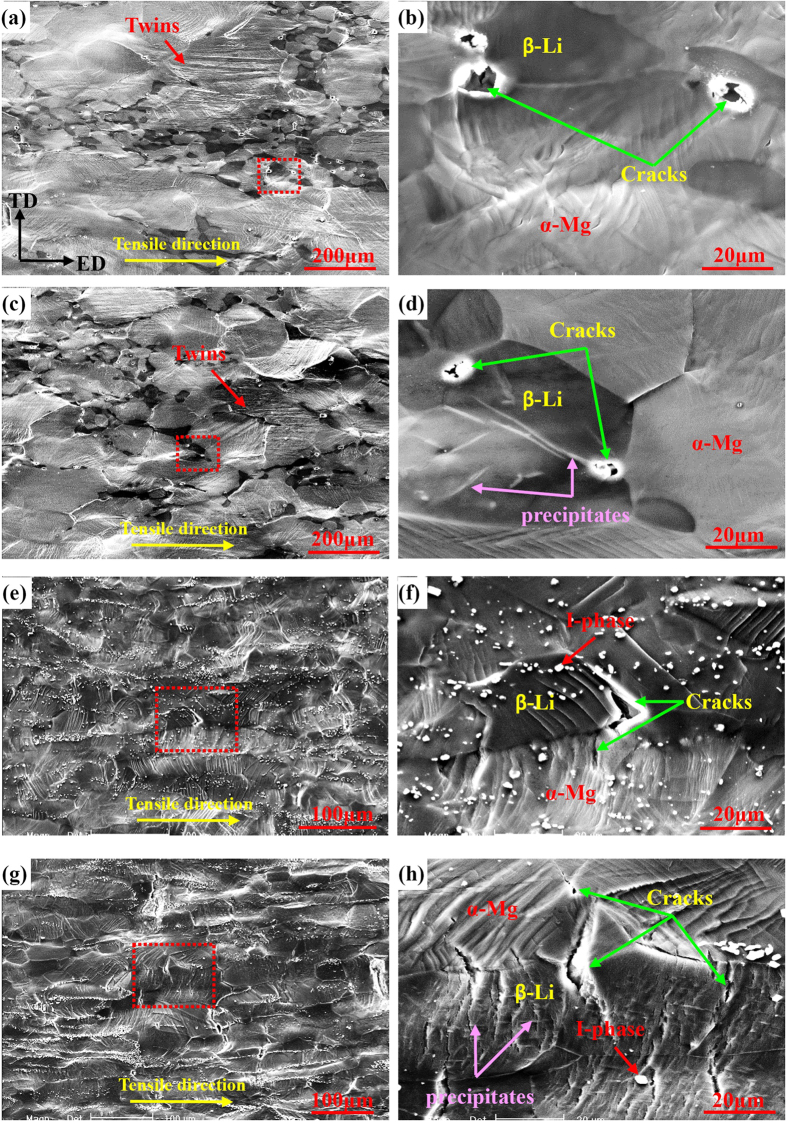
Observations to side surfaces near the fracture sites of: (**a**) and (**c**) Mg-6%Li alloy before and after natural ageing for 35 days, (**e**) and (**g**) Mg-6%Li-6%Zn-1.2%Y alloy before and after natural ageing for 35 days. Images (**b**,**d**,**f** and **h**) are high magnification observations to the squared areas in images (**a**,**c**,**e** and **g**), respectively.

To reflect and compare the deformation mechanisms of two alloys before and after natural ageing for 35 days, the microstructure evolutions of side surfaces with a distance of about 1 mm from the fracture sites were observed, as shown in Fig. 8. For the solution treated Mg-6%Li alloy, twins and slip bands can be observed (Fig. 8(a)). High-magnified observation reveals that micro cracks can mainly nucleate at α-Mg/β-Li interfaces (Fig. 8(b)). After ageing treatment, similar deformation mechanism and micro cracks can be observed (Fig. 8(c–d)). Since the hardness value of α-Mg phases is lower than that of β-Li phases (Fig. 6(a)), more slip bands and twins can occur in α-Mg phases (Fig. 8(a–d)). Moreover, β-Li phases (bcc structure) have enough independent slip systems and α-Mg phases (hcp structure) have limited independent slip systems, the stress concentration at α-Mg/β-Li interfaces can be easily induced due to the incompatible plastic deformation between two phases. Then, micro cracks will preferentially occur at α-Mg/β-Li interfaces.

For the Mg-6%Li-6%Zn-1.2%Y alloy, slip bands can be widely formed on the sample surfaces, but deformation twinning can be hardly activated before and after ageing treatment (Fig. 8(e–g)). Due to the higher volume fraction of β-Li phases, the macro plastic deformation can be well accommodated and basically no activation of twinning is needed. Similarly, the hardness value of α-Mg phases is much lower than that of β-Li phases of Mg-6%Li-6%Zn-1.2%Y alloy before ageing treatment (Fig. 6), resulting in the formation of dense slip bands in α-Mg phases (Fig. 8(f)). However, due to the increased hardness value of α-Mg phases after ageing treatment, dense slip bands can be formed in both α-Mg and β-Li phases (Fig. 8(h)). Then, micro cracks preferentially occur at these slip bands. Moreover, cracks can also nucleate at α-Mg/β-Li interfaces.

In summary, the volume fraction of β-Li phases in Mg-6%Li alloy decreases after solid solution treatment. However, the addition of Zn and Y can remarkably improve the thermal stability of β-Li phases in Mg-6%Li-6%Zn-1.2%Y alloy. Precipitation of needle-shaped α-Mg particles can occur in both two alloys at room temperature. Due to the formation of abundant MgLiZn precipitates, the growth of α-Mg precipitates in Mg-6%Li-6%Zn-1.2%Y alloy can be suppressed. For the Mg-6%Li-6%Zn-1.2%Y alloy, the quick precipitation of tiny MgLiZn particles is the dominant factor for causing the age-softening response of β-Li phases. For the Mg-6%Li alloy, cracks preferentially nucleated at the α-Mg/β-Li interfaces during tensile process. However, for the Mg-6%Li-6%Zn-1.2%Y alloy, micro cracks can occur at the α-Mg/β-Li interfaces and slip bands in α-Mg and β-Li phases.

## Methods

The materials used in the present work were the as-extruded Mg-6%Li and Mg-6%Li-6%Zn-1.2%Y (wt.%) alloy plates with a thickness of 15 mm and a deformation ratio of 5, which were prepared at the Magnesium Alloy Research Department, Institute of Metal Research, Chinese Academy of Sciences, Shenyang, China. Previous work demonstrated that the Mg-6%Li alloy was composed of α-Mg and β-Li phases, whereas the main phases in the Mg-6%Li-6%Zn-1.2%Y alloy were α-Mg, β-Li, I-phase, MgLiZn and W-phase^14^,^16^.

For the I-phase strengthened Mg-6%Li-6%Zn-1.2%Y alloy, samples were performed a stepped solid solution treatment at 330 °C for 2 h and 400 °C for 1 h in an air furnace. For comparison, samples of Mg-6%Li alloy were performed a single step solid solution treatment at 400 °C for 1 h to minimize the excessive reduction in volume fraction of β-Li phases at elevated temperature^14^. After annealing, samples were quenched into water at room temperature. To avoid the possible burning during solid solution treatments, samples were covered with graphite powders and then wrapped with a number of Al foil layers. Subsequently, samples were naturally aged. Then, samples were ground with SiC paper up to 5000 grit and finely polished to a 1 μm finish with ethanol. Microstructure was observed by using optical microscopy (OM), scanning electron microscopy (SEM; XL30-FEG-ESEM) equipped with energy dispersive X-ray spectroscopy (EDS) and transmission electron microscopy (TEM; JEOL2100F) in conjunction with energy dispersive X-ray spectroscopy (EDS). Thin foil specimens for TEM observations were prepared by mechanical thinning and subsequent argon ion milling at −70 °C. To reveal the grain structure of two alloys before and after solid solution treatments, the as-polished surfaces were etched with 4% nitric acid + 96% ethanol. Then, the average grain size was determined by using the mean linear intercept method.

Micro Vickers hardness was measured by using a LECO LM-247AT Hardness Tester with a load of 200 g (HV_0.2_) and a duration time of 15 s. Tensile samples were machined into a gauge with a dimension of 25 mm in length, 6 mm in width and 3 mm in thickness. The axial direction of tensile samples was parallel to the extruded direction (ED) of the plate. Tensile experiments were conducted on a MTS (858.01 M) tester at a constant strain rate of 1 × 10^−3^ s^−1^ at room temperature. To ensure the reproducibility, at least replicate measurements were carried out for each condition. After testing, side surfaces near the fracture sites were observed by using SEM.

## Additional Information

**How to cite this article**: Li, C. Q. *et al*. Natural ageing responses of duplex structured Mg-Li based alloys. *Sci. Rep.* **7**, 40078; doi: 10.1038/srep40078 (2017).

**Publisher's note:** Springer Nature remains neutral with regard to jurisdictional claims in published maps and institutional affiliations.

## Figures and Tables

**Table 1:** Tensile properties of the Mg-6%Li and Mg-6%Li-6%Zn-1.2%Y alloys before and after natural ageing treatment for 35 days.

Alloys	Conditions	YS (MPa)	UTS (MPa)	EL (%)
Mg-6%Li	Solution treated	80 ± 5	140 ± 3	27 ± 2
	Ageing treated	77 ± 4	133 ± 4	30 ± 3
Mg-6%Li-6%Zn-1.2%Y	Solution treated	183 ± 3	252 ± 5	20 ± 2
	Ageing treated	171 ± 5	234 ± 5	27 ± 3
